# Evaluation and Comparison of Stresses Between All-on-4 and All-on-6 Treatment Concepts With Three Different Prosthetic Materials in the Maxilla: A Finite Element Analysis Study

**DOI:** 10.7759/cureus.71362

**Published:** 2024-10-13

**Authors:** Sneha B Jaiswal, Shashikala Jain, Vaibhav Jain, Ramanjeet K Grover, Amit A Kale, Lakhan Talreja

**Affiliations:** 1 Department of Prosthodontics, Maharaja Ganga Singh Dental College and Research Centre, Sri Ganganagar, IND; 2 Department of Computer Science and Electronics, Indian Institute of Technology Dhanbad, Dhanbad, IND; 3 Department of Oral and Maxillofacial Surgery, Maharaja Ganga Singh Dental College and Research Centre, Sri Ganganagar, IND

**Keywords:** cortical bone, finite element analysis, implant, prostheses, stresses

## Abstract

Introduction: This study aimed to evaluate the comparative analysis of stresses on and around implants and surrounding cortical bone using three different prosthetic materials in hybrid prosthesis using the all-on-4 technique and all-on-6 technique in the maxilla using finite element analysis (FEA).

Materials and methods: An in vitro FEA study was conducted where a three-dimensional (3D) model of an edentulous maxilla and four different models of the mandible were built using ANSYS software version 18 (Ansys, Inc., Canonsburg, Pennsylvania, United States). Group A comprised the all-on-4 technique where two vertical implants were placed in the lateral incisor area, and two tilted implants at 30^o ^were placed in the second premolar area, whereas group B included the all-on-6 technique, where four vertical implants were placed in the central incisor and canine area, along with two tilted implants in second premolar area. Subsequently, prosthetic materials such as acrylic, porcelain fused to metal (PFM), and zirconia were studied. Four different opposing arch materials were made: natural teeth (A1 and B1), complete dentures (A2 and B2), implant-supported overdentures (A3 and B3), and hybrid prosthesis (A4 and B4). The von Mises stresses were recorded at the implant and cortical bone to summarize the effect of occlusal loads on the maxillary bone using the all-on-4 and all-on-6 techniques.

Results: In the all-on-4 technique, on evaluating stresses on implants, it was revealed that acrylic consistently demonstrated lower stress magnitudes in comparison to zirconia and PFM. The stress levels escalate from 38.72 MPa in model A1 to 72.43 MPa in model A4. Zirconia endured the most substantial stresses, ranging from 53.64 MPa (A1) to 84.87 MPa (A4). Similar results were obtained for the stresses on the cortical bone and for the all-on-6 technique. However, the stresses were lower in the all-on-6 technique than in the all-on-4 technique. The lowest stress values across all prosthetic materials were recorded when the maxillary arch was restored with acrylic teeth along with a titanium bar opposing natural dentition.

Conclusion: The least stresses were noticed in the all-on-6 technique with acrylic prosthetic material opposing the natural dentition. As the all-on-6 treatment concept showed the most favorable biomechanical behavior, it can be considered a viable alternative for moderate atrophic maxillary rehabilitation.

## Introduction

Dental implantology has fundamentally transformed the discipline of prosthodontics, affording patients a reliable and effective treatment for the absence of teeth. A notable progression within this domain is the introduction of the "All-on-4" and "All-on-6" methodologies, which facilitate comprehensive arch restoration utilizing a reduced number of implants [1]. These methodologies facilitate comprehensive dental rehabilitation by utilizing prosthetic frameworks anchored on four or six optimally positioned implants within either the maxillary or mandibular regions. The installation of these implants frequently necessitates angling of the posterior implants to circumvent critical anatomical formations, such as the maxillary sinuses or the inferior alveolar nerve, thereby enhancing osseointegration and the overall stability of the implants. Nevertheless, the biomechanical response of tilted implants, along with their adjacent osseous structures, under masticatory loads continues to be an essential domain of research, particularly with respect to the distribution of stress [2].

Finite element analysis (FEA) has emerged as a sophisticated analytical methodology for modeling and predicting the biomechanical behavior of implants and adjacent osseous structures in diverse clinical contexts. This analytical approach enables researchers to investigate the impact of various mechanical forces produced during mastication or other functional activities on both the implant and surrounding bone within a three-dimensional (3D) framework. Through the simulation of authentic physiological conditions within a regulated virtual setting, FEA offers invaluable perspectives on the distribution of stress across the implant-bone interface, which is paramount for ensuring long-term efficacy and mitigating complications, such as bone resorption or implant failure [3].

High stress around implants can lead to a range of clinical consequences. Excessive stress can result in bone resorption, implant failure, or damage to surrounding tissues. Over time, these stresses can cause microfractures or compromise the osseointegration process, where the implant is supposed to bond with the bone. Material selection for prostheses plays a critical role in mitigating these stresses. To prevent these issues, materials such as zirconia, composite resins, acrylic, and biocompatible polymers are being explored to better match the mechanical properties of bone, reduce stress concentrations, and improve long-term outcomes.

The selection of prosthetic materials utilized in hybrid prostheses is a pivotal element that affects the distribution of mechanical stress. Frequently employed materials in hybrid prostheses include acrylic, zirconia, and porcelain fused to metal (PFM), each of which is characterized by unique mechanical attributes such as the modulus of elasticity and fracture toughness. These attributes significantly impact the manner in which stresses are conveyed from the prosthesis to the underlying implants and osseous structure [4]. A thorough comparative analysis of these materials using FEA could yield significant insights for the informed selection of the most appropriate prosthetic material for hybrid prostheses supported by the all-on-4 or all-on-6 methodologies.

Consequently, the present investigation was designed to assess and compare the distribution of stress surrounding implants and adjacent osseous structures by utilizing the all-on-4 and all-on-6 methodologies in the maxillary region, incorporating acrylic, zirconia, and PFM prosthetic materials within a 3D FEA framework.

## Materials and methods

Study design and setting

This in vitro study was conducted at the Department of Prosthodontics, Maharaja Ganga Singh Dental College and Research Centre, Sri Ganganagar, Rajasthan, India, from October 2022 to February 2024. Since this was a simulation study based on 3D models, no ethical approval was required. The study design followed these steps: 3D modeling of the maxilla and mandible, where a geometric model of an edentulous maxillary and four different models of mandibular bone were created based on standard human anatomy; second, the implant and prostheses were designed for both the all-on-4 and all-on-6 techniques; and third, the three commonly used materials were tested: acrylic, zirconia, and PFM. The final model was subjected to FEA simulations under static masticatory loading. The stress distribution and magnitude were recorded and compared across the different configurations and materials.

3D model

A 3D model of the edentulous maxilla and four different models of the mandible were constructed based on the CT scan data from an adult human subject. The CT data were converted into a 3D surface model using ANSYS software version 18 (Ansys Inc., Canonsburg, Pennsylvania, United States). The maxilla was modeled to include both 1 mm cortical and 2 mm cancellous bone with 2 mm of thick mucosa with accurate anatomical representation of bone thickness and density.

Implant and prosthetic models

**Figure 1:**
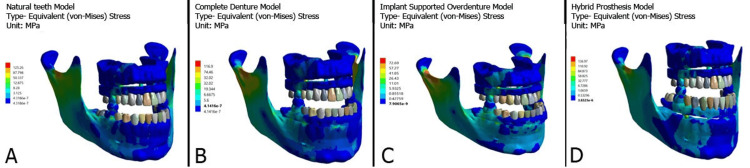
All-on-4 implant system in maxillary arch opposing four different mandibular prostheses: (A) Natural teeth, (B) Complete denture, (C) Implant supported overdenture, (D) Hybrid prosthesis. MPa: Megapascal

**Figure 2:**
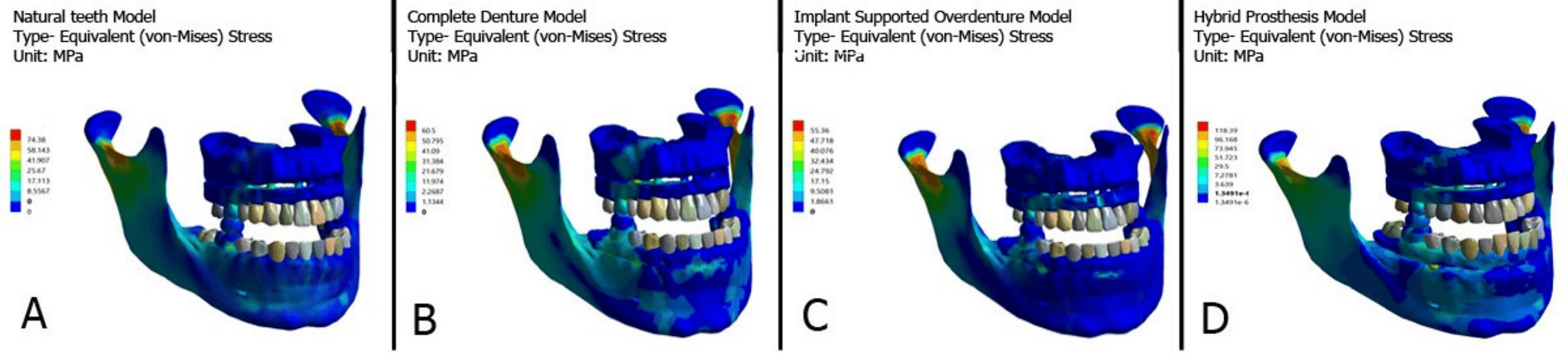
All-on-6 implant system in maxillary arch opposing four different mandibular prostheses: (A) Natural teeth, (B) Complete denture, (C) Implant supported overdenture, (D) Hybrid prosthesis. MPa: Megapascal

Two different implant configurations corresponding to the all-on-4 and all-on-6 techniques were modeled based on standard commercially available dental implants with a length of 13 mm and a diameter of 4.3 mm (Dentium Co. Ltd, Seoul, South Korea). All implants were made of grade 5 titanium (Ti) alloy (Ti-6Al-4V). For the all-on-4 configuration, the two anterior implants were placed vertically in the lateral incisor region, and the two posterior implants were placed in the second premolar region, which was tilted at an angle of 30° in the posterior region. For the all-on-6 configuration, four anterior implants were placed vertically (two in the central incisor area and two in the canine area) and two posterior implants were tilted at the same angle as in the all-on-4 setup. Anterior implants were fitted with straight multi-unit abutments, whereas posterior implants were fitted with angled multi-unit abutments (Dentium Co. Ltd). Three different materials were tested in the maxillary arch: acrylic framework with Ti bar, zirconia framework with Ti bar, and PFM framework with Ti bar. To oppose this maxillary arch, four different models of the mandible were created (natural dentition, complete denture, implant-supported overdenture, and hybrid prosthesis). Therefore, to assess the stress distribution, 24 3D FEA models were created. The nodes and elements of various models with model names are shown in Table 1, whereas the FEA models of the acrylic framework in the maxillary arch opposing four different models in the mandible are shown in Figure 1 and Figure 2.

**Table 1:** Finite element analysis (FEA) models.

Model	S.No.	Model name	Number of elements	Number of nodes
All-on-4	A1	Maxillary arch restored with titanium bar opposed by natural teeth	451458	762075
	A2	Maxillary arch restored with titanium bar opposed by complete denture	454597	756954
	A3	Maxillary arch restored with titanium bar opposed by implant supported overdenture	517900	867992
	A4	Maxillary arch restored with titanium bar opposed by hybrid prosthesis	455219	782843
All-on-6	B1	Maxillary arch restored with titanium bar opposed by natural teeth	481564	818241
	B2	Maxillary arch restored with titanium bar opposed by complete denture	484832	813677
	B3	Maxillary arch restored with titanium bar opposed by implant supported overdenture	549028	927508
	B4	Maxillary arch restored with titanium bar opposed by hybrid prosthesis	514362	878287

FEA setup

The 3D models of the maxilla, implants, and prostheses were imported into ANSYS software. The models were discretized into finite elements using tetrahedral mesh elements. A finer mesh was applied to the implant-bone interface to ensure accurate stress analysis in this critical area. A mesh convergence analysis was performed to ensure that further refinement of the mesh did not significantly change the stress results. The material properties of the cortical and cancellous bone, implants, and prosthetic materials were assigned based on the values reported in the literature (Table 2).

**Table 2:** Mechanical properties of the materials. GPa: Gigapascal

Material	Young's modulus (GPa)	Poisson's ratio
Cortical bone	14.1	0.30
Cancellous bone	1.37	0.30
Mandibular disc	44.1	0.44
Dentin	18.6	0.32
Enamel	84	0.32
Mucosa	1	0.37
Periodontal ligament	68.9	0.45
Acrylic resin	2.2	0.31
Titanium alloy	105	0.37
Zirconia	210	0.30
Porcelain fused to metal	145	0.30
Food bolus	84.1	0.33

The materials were assumed homogenous, isotropic, and linearly elastic. The contact conditions between the implant and bone as well as between the prosthesis and implants were defined as bonded, assuming no micromovements between these interfaces. This represents an ideal clinical scenario in which osseointegration can be achieved.

The boundary conditions were set as follows: the base of the maxillary bone was fixed to prevent movement, simulating the clinical situation in which the maxilla is supported by the surrounding skull structures. This ensured that the displacement occurred only in response to the applied loads. For each model, an occlusal load was delivered to the first molar area using a spherical solid substance (10 mm in diameter) to simulate foodstuffs. Masticatory forces were emulated by exerting a static vertical load of 150 N, uniformly distributed across the occlusal surfaces of the prosthesis. This load corresponded to the mean bite force in the molar area. Furthermore, oblique forces of 100 N were introduced at an angle of 30° to replicate lateral masticatory forces. These loading scenarios aimed to replicate the complex nature of the forces acting on the prosthesis during normal chewing. For each configuration (all-on-4 and all-on-6) and material, the stress distributions were calculated.

The von Mises stress was used to evaluate the magnitude of the stresses in the bones and implants. This criterion is commonly used to assess whether materials yield under a given loading condition. The stress distribution in the peri-implant bone and prosthesis was assessed to determine the areas of stress concentration. Special attention was given to the bone-implant interface and the effect of different prosthetic materials on this distribution. The results of the FEA simulations were compared for different implant configurations.

## Results

Table 3 illustrates the von Mises stress values, measured in megapascals (MPa) for Ti bars and implants utilized within an all-on-4 dental implant framework.

**Table 3:** von Mises stress at titanium (Ti) bar and implants in all-on-4 model. FEA: Finite element analysis; MPa: Megapascal

FEA model	Restorative material	von Mises stresses at Ti bar and implants (MPa)
A1	Acrylic	38.72
	Zirconia	53.64
	Porcelain fused to metal	50.33
A2	Acrylic	54.62
	Zirconia	74.46
	Porcelain fused to metal	61.59
A3	Acrylic	54.6
	Zirconia	72.69
	Porcelain fused to metal	65.95
A4	Acrylic	72.43
	Zirconia	84.87
	Porcelain fused to metal	79.04

The findings were delineated according to the prosthetic materials employed (acrylic, zirconia, and PFM) across four distinct FEA models (A1 to A4). Acrylic consistently demonstrated lower stress magnitudes than zirconia and PFM. The stress levels escalate from 38.72 MPa in model A1 to 72.43 MPa in model A4. Zirconia endured the most substantial stresses, ranging from 53.64 MPa (A1) to 84.87 MPa (A4), signifying that this material was subjected to enhanced stress under analogous conditions. PFM exhibited intermediate stress values, spanning from 50.33 MPa (A1) to 79.04 MPa (A4), generally exceeding those of acrylic yet falling short of those of zirconia. As the FEA model transitioned from A1 to A4, an increase in the von Mises stress values was observed for all prosthetic materials. This indicated that the lowest stress values across all prosthetic materials were recorded when the maxillary arch was restored with a titanium bar opposing the natural dentition, whereas the highest stress values were detected when opposing a hybrid prosthesis in the mandibular arch, implying that more intricate or demanding load scenarios contribute to elevated stress on the implants and bars. Similar results were noted when stresses were studied on the cortical bone with the highest stresses in A4 and zirconia. The lowest stresses were observed with the acrylic material and A1 (Table 4).

**Table 4:** von Mises stress at cortical bone in all-on-4 model. FEA: Finite element analysis; MPa: Megapascal

FEA model	Restorative material	von Mises stresses at cortical bone (MPa)
A1	Acrylic	92.71
	Zirconia	125.26
	Porcelain fused to metal	96.37
A2	Acrylic	101.59
	Zirconia	116.9
	Porcelain fused to metal	105.69
A3	Acrylic	68.97
	Zirconia	70.75
	Porcelain fused to metal	68.97
A4	Acrylic	112.36
	Zirconia	136.97
	Porcelain fused to metal	124.59

In the all-on-6 dental implant framework, von Mises stress metrics, expressed in MPa, elucidate the response of various prosthetic substances to different load scenarios. The stress metrics were documented for three prosthetic substances (acrylic, zirconia, and PFM) across four FEA models (B1-B4). These metrics reflect the stress exerted on the cortical bone that supports the prosthetic devices. For acrylic, the stress spans from 55.36 MPa in model B2 to 109.73 MPa in B4. Acrylic consistently exhibited lower stress levels than the other materials, although there was a notable increase in stress under more rigorous conditions, particularly in B3 and B4. This finding implies that, while acrylic may be advantageous in low-stress environments, its durability may be compromised under elevated stress conditions. Zirconia demonstrated the highest stress values across all FEA models, with stress levels commencing at 60.5 MPa in B2 and escalating to 118.39 MPa in B4. The stress metrics for this material suggested that it encountered substantial demands and experienced greater strain, particularly under the intricate loading scenarios in B3 and B4. The capacity of zirconia to endure such stresses could be beneficial in more severe conditions, although it also indicated that the material was subjected to heightened mechanical forces. PFM revealed intermediate stress values, ranging from 58.93 MPa in B2 to 110.83 MPa in B4. Its stress metrics were generally superior to those of acrylic but inferior to those of zirconia, signifying that it offered a balanced performance in terms of durability and stress absorption. In summary, the stress values for all materials exhibited an upward trend from B1 to B4, indicating an increase in load conditions or complexity. The elevated stress values observed in B3 and B4 imply that the implants and prosthetic materials experienced increased strain in these contexts (Table 5).

**Table 5:** von Mises stress at cortical bone in all-on-6 model. FEA: Finite element analysis, MPa: Megapascal

FEA model	Restorative material	von Mises stresses at cortical bone (MPa)
B1	Acrylic	62.98
	Zirconia	74.38
	Porcelain fused to metal	68.47
B2	Acrylic	55.36
	Zirconia	60.5
	Porcelain fused to metal	58.93
B3	Acrylic	89.67
	Zirconia	98.37
	Porcelain fused to metal	93.59
B4	Acrylic	109.73
	Zirconia	118.39
	Porcelain fused to metal	110.83

Similar results were noted when stresses were evaluated at the Ti bar and implant bone, with the highest stresses in B4 and zirconia. The lowest stresses were observed with the acrylic material and B1 (Table 6).

**Table 6:** von Mises stress at titanium (Ti) bar and implants in all-on-6 model. FEA: Finite element analysis; MPa: Megapascal

FEA model	Restorative material	von Mises stresses at Ti bar and implants (MPa)
B1	Acrylic	29.08
	Zirconia	41.9
	Porcelain fused to metal	35.66
B2	Acrylic	41.01
	Zirconia	55.68
	Porcelain fused to metal	47.71
B3	Acrylic	45.2
	Zirconia	73.07
	Porcelain fused to metal	62.92
B4	Acrylic	91.72
	Zirconia	96.16
	Porcelain fused to metal	92.7

## Discussion

The current investigation was undertaken to evaluate von Mises stress on Ti bars and implants within all-on-4 and all-on-6 dental implant frameworks, yielding significant insights into the biomechanical properties of various prosthetic substances, such as acrylic, zirconia, and PFM, under diverse loading conditions.

Stress distribution in all-on-4 and all-on-6 model

In the all-on-4 configuration, the stress measurements on the cortical bone and implants were consistently inferior to those recorded in the all-on-6 configuration across all the prosthetic materials examined. This finding corroborates prior investigations that indicated that the quantity of implants influences stress distribution, with a reduced number of implants (four in this instance) resulting in diminished stress values on the individual components [5,6]. This finding is supported by Pandey et al. [7]. The presence of distal implants in the all-on-6 system provides better stress distribution over a larger area, creating less stress on the cortical bone.

The results of our study further revealed that acrylic demonstrated the lowest von Mises stress values across all the tested combinations. These comparatively low-stress values imply that acrylic is an acceptable prosthetic material under moderate load conditions, as substantiated by existing literature indicating that acrylic offers sufficient shock absorption and load distribution within analogous implant systems [6]. Nevertheless, as the load intensifies, the stress imposed on the acrylic increases, particularly in contrast to the hybrid prosthesis, suggesting that it may not be appropriate for high-load situations. This phenomenon may be attributed to the elastic modulus of the acrylic resin (2.2 GPa), which is significantly lower than that of both ceramic (82 GPa) and zirconia (210 GPa) [8]. This disparity allows enhanced energy absorption and reduced stress transmission to the underlying osseous tissue. Similar findings were reported in the previous studies [6,8].

Zirconia, however, demonstrated the highest stress values among the materials. This result is consistent with studies indicating that zirconia, while highly durable, is more rigid than acrylic and tends to absorb less load, leading to higher stresses on the implant. The higher stress in zirconia suggests that it may be more prone to mechanical failure under extreme conditions, which should be considered when used in patients with heavy occlusal forces [9]. Our findings are in line with those of previous studies in which the highest stresses were noted with zirconia [10,11].

PFM showed intermediate stress values, with von Mises stresses ranging from 50.33 MPa (A1) to 79.04 MPa (A4). This material provides a balance between durability and load absorption, as highlighted in the literature, which praises its long-term success in prosthetic restorations [12]. The material’s stress values suggested that it could be an optimal choice for patients requiring a balance between strength and flexibility.

It was further noticed in our study that the lowest stresses were noticed on the cortical bone and implants when the maxillary prosthesis opposed the natural dentition, and the highest stresses were noticed when it opposed the hybrid prosthesis. This could be due to the fact that natural dentition exhibits a more equitable distribution of occlusal forces owing to its intrinsic dynamic and adaptive properties. Over an extended period, these teeth experience progressive wear, which contributes to the maintenance of harmonious occlusion, while their contact surfaces (comprising cusps and grooves) are structurally engineered to efficiently distribute occlusal loads. This dynamic alteration alleviates the peak forces encountered during mastication. In contrast, hybrid prostheses are primarily constructed from more rigid materials such as metal-acrylic or metal-ceramic composites. These materials demonstrate enhanced rigidity and are comparatively less proficient at uniformly distributing occlusal forces compared to natural dentition. Moreover, the occlusal surfaces of prosthetic teeth exhibit a deficiency in adaptive characteristics, resulting in occlusal contacts that may become increasingly intense and localized, thereby producing elevated peak forces on the implants and adjacent osseous structures [13,14].

Comparative analysis of all-on-4 and all-on-6 models

Comparing the all-on-4 and all-on-6 models revealed that adding two more implants significantly decreased the von Mises stresses for all materials on the cortical bone and implants. This finding was supported by earlier studies that suggested that an increase in the implant number should reduce stress [7,15]. For acrylic, the increase in stress between the two models was considerable, especially in the more demanding load conditions of the opposing hybrid prosthesis. This suggested that acrylic may not be the best option for full-arch restorations under high-load conditions, as supported by studies indicating its limited durability [6,8]. In contrast, the higher stress values of zirconia in both models suggested that it is better suited for patients with high occlusal forces, although it might lead to mechanical wear on the implants over time [9]. The moderate stress values for PFM in both models indicated that it was a durable and balanced choice [13]. It has been concluded in a systematic review by Reddy et al. that comparative results from 3D FEA studies showed that 3D FEA, when matched with in-vivo strain gauge measurements corresponds with clinical outcomes [3]. A study by Aboelfadl et al. concluded that the combination of fixed implant prosthesis without cantilever using a rigid zirconia material exhibits better biomechanical behavior and stress distribution around bone and implants [16]. According to Sirandoni et al., it was observed that polyether ether ketone and acrylic resin frameworks showed the highest total deformation values, showing decreases of von Mises stresses in the frameworks, implants, and abutments, but with high tensile stress in the trabecular bone that achieved critical values [17].

Clinical implications

This investigation underscores that opposing hybrid prostheses apply greater concentrated stresses on implants and cortical bone than natural dentition, thereby amplifying the potential for implant fatigue, osseous resorption, and mechanical failure. Practitioners should consider material characteristics and occlusal forces to enhance the durability of implants and reduce complications associated with full-arch rehabilitation. All-on-6 implant system with acrylic is a good choice for elderly patients with poor bone quality, whereas zirconia is better for younger patients with good bone quality. PFM is usually not indicated as it chips off easily under heavy masticatory load.

Limitations

The constraints inherent in this FEA investigation include the reduction of intricate biological architectures, including the anisotropic characteristics of bone, and the omission of soft tissues, such as the periodontal ligament, which is critical in the force dissipation mechanisms observed in natural dentition. The model further presupposes idealized and homogeneous material properties along with consistent loading conditions, which may inadequately represent the heterogeneity present in clinical environments. Moreover, individualized factors pertaining to patients, such as bone quality, implant positioning, and unique occlusal forces, were not integrated into the analysis. Consequently, these simplifications restrict the applicability of the findings to real-world scenarios that are specific to individual patients. The absence of patient-specific factors (such as individual occlusal forces, bone quality, and implant positioning) may affect the real-world applicability of the FEA results. Additionally, the exclusion of dynamic loading or temperature fluctuations in FEA models may affect material performance, and therefore, their responses in real-world scenarios. 

## Conclusions

The results of the present study illustrated that opposing hybrid prosthetic devices to implant-supported prostheses imposes greater von Mises stress on both implants and cortical bone in comparison to natural dentition, especially in all-on-4 configurations. All-on-6 implant system demonstrated less stress than the all-on-4 system. Acrylic materials exhibited comparatively low stress levels, whereas zirconia endured the most significant stress. These results inform the selection of materials and design of implants aimed at enhancing the durability of prosthetics and mitigating potential complications.
